# The effect of age on cognitive performance of frontal patients

**DOI:** 10.1016/j.neuropsychologia.2015.06.011

**Published:** 2015-08

**Authors:** Lisa Cipolotti, Colm Healy, Edgar Chan, Sarah E. MacPherson, Mark White, Katherine Woollett, Martha Turner, Gail Robinson, Barbara Spanò, Marco Bozzali, Tim Shallice

**Affiliations:** a Department of Neuropsychology, National Hospital for Neurology and Neurosurgery, London, UK; b Dipartimento di Psicologia, Università di Palermo, Italy; c Centre for Cognitive Ageing and Cognitive Epidemiology, Human Cognitive Neuroscience, Department of Psychology, University of Edinburgh, Edinburgh, UK; d Department of Neuroradiology, National Hospital for Neurology and Neurosurgery, London, UK; e Institute of Cognitive Neuroscience, University College London, UK; f Wolfson Neurorehabilitation Unit, Queen Mary’s Hospital, London, UK; g School of Psychology, The University of Queensland, Brisbane, Australia; h Neuroimaging Laboratory, Santa Lucia Foundation, Rome, Italy; i International School for Advanced Studies (SISSA-ISAS), Trieste, Italy

**Keywords:** PFC, prefrontal cortex, WMA, white matter abnormalities, TBI, traumatic brain injury, IQ, Intelligence Quotient, CVA, cerebrovascular accident, HC, healthy controls, NART, National Adult Reading Test, RAPM, Raven's Advanced Progressive Matrices, GNT, Graded Naming Test, IL, Incomplete Letters and, CWMA, Composite White Matter Abnormalities, Aging, Cognitive performance, Frontal lesions non-frontal lesions, Executive functions

## Abstract

Age is known to affect prefrontal brain structure and executive functioning in healthy older adults, patients with neurodegenerative conditions and TBI. Yet, no studies appear to have systematically investigated the effect of age on cognitive performance in patients with focal lesions. We investigated the effect of age on the cognitive performance of a large sample of tumour and stroke patients with focal unilateral, frontal (*n*=68), or non-frontal lesions (*n*=45) and healthy controls (*n*=52). We retrospectively reviewed their cross sectional cognitive and imaging data. In our frontal patients, age significantly predicted the magnitude of their impairment on two executive tests (Raven's Advanced Progressive Matrices, RAPM and the Stroop test) but not on nominal (Graded Naming Test, GNT) or perceptual (Incomplete Letters) task. In our non-frontal patients, age did not predict the magnitude of their impairment on the RAPM and GNT. Furthermore, the exacerbated executive impairment observed in our frontal patients manifested itself from middle age. We found that only age consistently predicted the exacerbated executive impairment. Lesions to specific frontal areas, or an increase in global brain atrophy or white matter abnormalities were not associated with this impairment. Our results are in line with the notion that the frontal cortex plays a critical role in aging to counteract cognitive and neuronal decline. We suggest that the combined effect of aging and frontal lesions impairs the frontal cortical systems by causing its computational power to fall below the threshold needed to complete executive tasks successfully.

## Highlights

• The effects of age on cognitive performance of frontal and non-frontal patients.
• Exacerbated executive impairment in frontal patients.
• Exacerbated executive impairment was evident by middle age in frontal patients.
• No exacerbated impairment in non-frontal patients on any task.

## 1 Introduction

It is well-recognised that healthy aging is associated with a decline in cognitive processes. Research has shown that self-initiated frontal ‘executive’ processes appear to be the most affected (Craik, 1986) with the greatest anatomical changes found in the prefrontal cortex (PFC) compared with other cortical regions (e.g., O’Sullivan et al., 2001; MacPherson et al., 2002; West et al., 2002). Specifically, healthy older individuals have reduced cortical volume, increased white matter abnormalities (WMA) and functional over- or under-activation have all been documented in the PFC compared with young individuals (e.g., Cabeza, 2002; Raz et al., 2005; Sullivan and Pfefferbaum, 2006; Fjell et al., 2009; Head et al., 2009). These structural abnormalities are correlated with poorer executive performance (e.g., Nagahama et al., 1997; Gunning-Dixon and Raz, 2003; Van Petten et al., 2004; Raz et al., 2007; Cardenas et al., 2011). For example, increased WMA and smaller anterior cingulate cortex volume are associated with poorer performance on the Stroop test and fluid intelligence tasks (e.g., Raz et al., 2007; Elderkin-Thompson et al., 2008, but see Salthouse (2011)).

Similarly, pathological aging research, involving individuals such as those with dementia or other neurological conditions, has shown a decline in executive performance together with structural and functional changes in the PFC (e.g., Pachana et al., 1996; Matsuo et al., 2008; Debette and Markus, 2010; for a review see Cabeza and Dennis (2013)). This decline is thought to reflect ‘accelerated normal aging’, a process resembling normal aging but occurring earlier and faster, as a result of brain pathology (Buckner, 2004). Accelerated cognitive aging has also been documented following traumatic brain injury (TBI; e.g., Corkin et al., 1989). For example, older Vietnam veterans with penetrating head injury lesions involving the frontal lobes (although not exclusively) showed a greater decline in IQ than younger veterans (Raymont et al., 2008). Moreover, the effect of aging on TBI patients' appears to specifically affect executive (Stroop test) performance but not verbal memory performance (Klein et al., 1996).

However, the non-specific nature of brain-related changes in neurodegenerative diseases and TBI limits our ability to draw firm conclusions that age moderates lesion-related impairment in executive functions. Examination of patients with more focalised frontal and non-frontal lesions such as those resulting from stroke and tumour would overcome such limitations. A meta-analysis has hinted that age disproportionately affects performance on executive tasks in patients with frontal lesions (Alvarez and Emory, 2006). However, this review included patients with less focal lesions due to TBI, epilepsy, gunshot wounds and encephalitis, and a surprisingly small number of stroke (*n*=14) and tumour patients (*n*=1).

Older stroke and tumour patients have consistently been associated with higher mortality rates, poorer functional outcomes and general cognitive decline (e.g., Appelros et al., 2003; Nakayama et al., 1994; Patel et al., 2002; Yoshii et al., 2008). In stroke age has been associated with greater executive impairment (e.g., Pohjasvaara et al., 2002; Oksala et al., 2009) together with WM abnormalities and/or cortical atrophy (e.g., Jokinen et al., 2005; Kooistra et al., 2014). In brain tumour, middle-aged (36–59 years) and older (60+ years) patients performed more poorly than younger patients (<35) on a test of ‘executive’ function and information processing (Trail Making B; Kaleita et al., 2004; however, see Chan et al. (2014)). However, given the absence of a healthy control sample, it remains unclear whether the executive impairments in stroke and tumour reflect typical age-related decline or an exacerbated executive impairment. Furthermore, in these studies, the lesions in the stroke and tumour patients were not restricted to specific cortical areas. Thus, the high degree of variability in the patients' cognitive performance inevitably reduces one's ability to draw conclusions regarding the interaction between age, brain lesion and executive functioning.

To the best of our knowledge, no previous study has systematically examined the effects of age on cognitive performance in patients with focal lesion. The aim of our study was to investigate the effect of age on ‘executive’, nominal and perceptual tasks in a large sample of patients with focal, unilateral, frontal or non-frontal lesions and healthy controls. Structural data included the classification of frontal lesions in 4 major anatomical areas, measures of global brain atrophy and WM abnormalities.

## 2 Methods

### 2.1 Participants

Data from 122 patients who had attended the Neuropsychology Department of the National Hospital for Neurology and Neurosurgery, Queen Square, London, were retrospectively screened for study eligibility. All patients had a unilateral lesion confined to the frontal or non-frontal brain regions resulting from a cerebrovascular accident (CVA; stroke) or a brain tumour. All tumour patients had undergone tumour resection prior to neuropsychological assessment. Our exclusion criteria were as follows: i) age at the time of cognitive testing >80 years due to the availability of age matched healthy control data and standardised age norms for patients up to 80 years, ii) current or previous psychiatric disorders, iii) previous neurological disorders including CVAs or tumours, iv), presence of metastatic tumours, v) previous chemotherapy, vi) gross visual (i.e., cortical blindness), perceptual (i.e., neglect; agnosia), motor (i.e., hemiplegia) or language (i.e., dysphasia) impairment, vii) previous head trauma, viii) history of alcohol or drug abuse, ix) no MRI or CT scan results available, x) no neuropsychological data available, and xi) a score below the 5th percentile on a test of general intelligence (WAIS-III, Wechsler, 1997; WAIS-R, Wechsler, 1981; or Raven's Matrices, Raven, 1976). Non-native English speakers were only included in the study if they obtained a score at or above the 25th%ile on the National Adult Reading Test (NART, Nelson, 1982). This was to ensure that their English abilities were sufficient to cope with task demands.

Application of these exclusion criteria resulted in data from nine patients being removed (*n*=1 history of psychiatric disorder; *n*=3 history of neurological disorder; *n*=1 previous chemotherapy; *n*=1 hemiplegia; *n*=1 expressive dysphasia). Data from 68 frontal patients (38 males and 30 females) and 45 non-frontal patients (24 males and 21 females) were included in the study. The aetiologies of the frontal lesions were as follows: stroke (CVA, *n*=17); high-grade tumours (*n*=15); low-grade tumours (*n*=14); and meningioma (*n*=22). Thirty seven frontal patients had left hemisphere lesions and 31 had right hemisphere lesions. The mean time between damage and assessment for the frontal patients was 16.41 months (standard deviation (SD)=33.80 months). Five frontal patients had reported hemiparesis and 3 frontal patients had hemianopia. Other clinical and cognitive aspects of the frontal patients have been previously reported (MacPherson et al., 2010; Robinson et al., 2012, 2015; Murphy et al., 2013). Importantly for the current study, we have previously documented no significant differences in the performance of CVA, high- or low-grade tumour, or meningioma on the Raven's Advanced Progressive Matrices, Stroop Colour-Word and Graded Naming Tests. This suggests that the grouping together of frontal patients with different aetiologies is methodologically justifiable (Cipolotti et al., 2015).

The aetiologies of the non-frontal patients were as follows: stroke (CVA, *n*=13); high-grade tumours (*n*=10); low-grade tumours (*n*=10); and meningioma (*n*=12). Twenty-two non-frontal patients had left hemisphere lesions and 23 had right hemisphere lesions. The mean time between damage and assessment for the non-frontal patients was 17.40 months (SD=38.46 months). Three non-frontal patients had reported hemiparesis and 6 non-frontal patients had hemianopia.

Data from 52 healthy controls (HC) who did not significantly differ from the frontal and non-frontal patients in terms of age, gender, NART IQ and years of education were also reviewed (see Section 2.4.1). The study was approved by the National Hospital for Neurology and Neurosurgery and the Institute of Neurology joint Research Ethics Committee (UK).

### 2.2 Cognitive investigation

We retrospectively reviewed the cognitive performance of the patients and healthy controls on a single assessment comprising of well-known tests with published standardised normative data. For the frontal patients and healthy controls data was available on the following tests: National Adult Reading Test (NART; Nelson, 1982) used to estimate optimal pre-morbid functioning; the Raven's Advanced Progressive Matrices (RAPM; Arthur and Day, 1994)) which assesses non-verbal abstract reasoning; the Stroop test to assess response inhibition (Stroop; Trenerry et al., 1989); the Graded Naming Test (GNT; McKenna and Warrington, 1980) to assess nominal functions; and the Incomplete Letters test (IL; Warrington and James, 1991) to assess perceptual functions. Data for the NART, RAPM and GNT were available for all 68 frontal patients, while data for the IL test and Stroop test were available for 65 and 42 frontal patients respectively.

For the 45 non-frontal patients, data were available for the NART, RAPM and GNT. Data for the NART, RAPM, GNT, IL and Stroop were available for 52 HC (see test descriptions in S1 of the Supplementary materials). The listwise deletion method was used so no substitutions were made to the data. Missing values analyses were conduct (see Supplementary materials: S2). The results of these analyses satisfied the assumptions of Missing Completely at Random.

### 2.3 Neuroimaging investigation

For 62 out of 68 frontal patients, MRI (*n*=46) or CT scans (*n*=16) were available for analysis. One patient scan was excluded from the analysis due to movement artefacts. All scans were reviewed by two independent neurologists (MB and BS) who were blind to the medical history of each patient. Brain MRI scans were obtained on systems operated at 0.5, 1.5 or 3 T and included the acquisition of an axial dual-echo (DE), an axial fluid attenuated inversion recovery (FLAIR), and an axial and coronal T1-weighted scan. CT scans were obtained using spiral CT systems, with axial images acquired with an effective slice thickness of 5 mm and pitch of 1.5. Only T1-weighted MRI scans (or CT scans when MRI was not available) were used for the assessment of frontal lesions. DE and FLAIR images were used for the assessment of global brain atrophy and non-specific WMA.

For the non-frontal patients, only clinical neuroradiological reports based either on MRI or CT scans were available. This information was sufficient to localise the patients' unilateral lesion to the left or right posterior regions. No further analysis was possible.

#### 2.3.1 Investigation of the frontal lesions

The exclusion criteria and lesion assessment guidelines were based on detailed anatomical localisation methods using standard atlases (Duvernoy, 1991). All frontal lesions could only involve, and not extend beyond, the frontal lobe. The lesion localisation method is described in detail in Robinson et al. (2012). Briefly, each frontal patient was coded for the presence of lesion and oedema in each hemisphere in the anterior and posterior portion of 9 left and 9 right frontal subregions (18 subregions in total). A subregion was only coded as damaged if at least 25% was affected. To compare whether left and right frontal lobe lesions impact on cognitive performance differently, we merged the 9 left and the 9 right brain subregions and divided the patients into two groups: left and right frontal according to which hemisphere was damaged (see Section 2.4.2).

To investigate whether the number of patients with lesions in the different frontal areas varied, we employed the grouping method previously adopted by Stuss et al. (1998), Stuss at al. (2005), Murphy et al. (2013), MacPherson et al. (2010), Robinson et al. (2015) and Cipolotti et al. (2015). Lesions in the prefrontal subregions were grouped together to define the primary lesion site in one of four main areas: medial, left lateral, right lateral and orbitofrontal. For these four areas, the primary lesion site was defined as either a) damage restricted to the cortical subregions that defined the area, or b) damage affecting at least three cortical subregions used to define each area and no more than one other subregion (secondary site) belonging to an adjacent area. Patients with unilateral primary damage to the medial area had lesions in the left/right cingulate gyrus (anterior/posterior), left/right sub-genu, left/right medial and superior frontal gyrus (anterior/posterior). These frontal subregions correspond to Brodmann areas: 6, 8, 9, 10, 23, 24, 32 and 33. Patients with damage to the lateral areas (left or right) had lesions affecting the left or right lateral part of the superior frontal gyrus (anterior/posterior), the left or right middle frontal gyrus (anterior/posterior), and the left or right inferior frontal gyrus (anterior/posterior). These frontal subregions correspond to Brodmann areas 6, 8, 9 38, 44, 45, 46 and 47. Patients with damage to the orbitofrontal area had lesions in the left or right orbital cortex (Brodmann areas 10 and 11).

#### 2.3.2 Investigation of global brain atrophy

Visual quantification of global brain atrophy was assessed in frontal patients using FLAIR or CT scans, according to the method proposed by Scheltens et al. (1997). Scores ranged from: 0=absence of atrophy; 1=minimal atrophy; 2=moderate atrophy; and 3=severe atrophy. The consistency of the ratings between the two raters (MB and BS) and the internal consistency of one of the rater (BS) were assessed. Inter- and intrarater reliability coefficients were investigated using two-way mixed model inter/intra-class correlation (CC; McGraw and Wong, 1996). We found that the inter- and the Intra-CC were in the excellent range (inter-CC=.979; intra-CC=.979; Cicchetti, 1994). Only for a small subset of patients MRI and CT scans were available. The interreliability coefficient between MRI and CT scans was assessed as above. We found that the inter-CC was in the excellent range (inter-CC=.882).

#### 2.3.3 Investigation of white matter abnormalities (WMA)

To visually quantify WMA in frontal patients, we used the commonly used Fazekas' rating scale (Fazekas et al., 1987). It includes two sub-scales; periventricular WM abnormality (i.e., PVA sub-scale) and deep WM abnormality (i.e., DWMA sub-scale). We examined WMA using a combined composite score (CWMA) of these two subscales (i.e., the sum of the two scales; see Kearney-Schwartz et al. (2009) and Sanossian et al. (2011)). For MRI scans, WM abnormalities were defined as hyper-intense areas, detectable on DE and/or FLAIR images. For CT scans, WM abnormalities were defined as hypo-dense areas within the WM. Scores ranged from 0 – an absence of WM abnormalities, to 3 – the most severe degree of WM abnormalities. Similar to the global brain atrophy, the inter- and intra-rater reliability coefficients were investigated and were in the excellent range (PVA: Inter-CC=.956; Intra-CC=.958; DWMA: Inter-CC=.917; Intra-CC=.947). Again only for a small subset of patients MRI and CT scans were available. We found the Inter-CC was in the excellent range (PVA: Inter-CC=.857; DWMA: Inter-CC=1.0).

### 2.4 Statistical analyses

#### 2.4.1 Demographic and clinical analyses

To investigate whether the frontal and non-frontal patients and HC groups significantly differed in terms of age, NART IQ or years of education ANOVAs were conducted. To investigate whether there was a significant difference in terms of the gender ratio, a chi-square test was used. To examine whether frontal and non-frontal patients were matched for time between damage and assessment, *t*-test analysis was used. Laterality of lesion and the number of patients with hemiparesis and hemianopia were analysed using chi-square tests.

#### 2.4.2 Cognitive analyses

The cognitive scores for all tasks were assessed for normality, homogeneity of variance and outliers. We only found a significant difference in group variance for the Stroop test, with frontal patients demonstrating higher variance than controls. Therefore a squared transformation (*χ*^2^) was performed on the data.

To examine whether there was an effect of lesion laterality on cognitive performance, we investigated: (1) whether left and right unilateral frontal patients and healthy controls were matched at the time of testing for age, gender, NART IQ and years of education using ANOVAs and chi square test in the case of gender (see Supplementary material Table S1a), and (2) whether there was an effect of laterality on the cognitive performance of left and right unilateral frontal patients using ANCOVA and entering NART IQ and years of education as covariates. We found no effect of laterality hence we grouped left and right frontal patients into one frontal group (see S3 – for the cognitive performance of left and right frontal patients and healthy controls, in Supplementary material and Table S1b; for similar methodology, see Roca et al. (2010)).

In our primary analysis (The Effect of Age on Cognitive Performance), we analysed the effect of age using a procedure originally developed by Woolgar et al. (2010) to predict IQ deficits following frontal and parietal lesions. The authors estimated premorbid scores on general intelligence tests from a multiple regression equation derived from healthy controls, predicting IQ scores from patient's age and NART IQ. Following the same procedure, we used healthy control data to derive multiple regression equations to predict each patient's estimated premorbid score based on age, years of education and NART IQ, for each cognitive test (RAPM, Stroop, GNT and IL for frontal patients; RAPM, and GNT for posterior patients). Each patient's post-morbid score was then subtracted from his/her estimated premorbid score to produce a ‘discrepancy’ measure. A further linear regression analysis was then conducted for each test to investigate the relationship between age and discrepancy measures.

In a subsidiary analysis (Executive Performance across Three Age Groups), we followed the procedure used by Kennedy and Raz (2009) among others. We grouped our frontal patients and HC into younger (20–45 years), middle-aged (46–60 years) and older (61–80 years) age groups to further analyse executive performance. This follows standard clinical practise based on the presupposition that the effects of age on performance in patients may manifest in middle as well as in old age (e.g. Warrington, 1984; 1996; Baddeley et al., 1994; Wechsler, 1997; Cohn et al., 1984; Deary et al., 2009). It is also in line with evidence suggesting that thinning of the cortex occurs in middle age as well as old age (Salat et al., 2004) and that increased rate of white matter abnormalities begins in the fifth decade (Kennedy and Raz, 2009). To examine the demographic and clinical variables of our three age groups we used *t*-tests for age, chi-square test for gender and ANOVAs for NART and years of education (see Table S2 in Supplementary materials). The performance on the executive tasks was analysed using ANCOVAs, with participant group (frontal patients versus HC) and age group (younger, middle-aged and older) as independent variables. NART and years of education were entered as covariates. All post-hoc analyses were corrected for multiple comparisons (*α*=.017).

#### 2.4.3 Neuroimaging analysis

##### 2.4.3.1 Analysis of frontal lesions

Chi-square analyses were used to investigate whether there were significant differences in the number of patients with damage involving the four main frontal areas: medial, left lateral, right lateral and orbito-frontal. All post-hoc Chi-square analyses were corrected for multiple comparisons (*α*=.0125).

##### 2.4.3.2 Analysis of global brain atrophy

Linear regression analysis was used to investigate the relationship between global brain atrophy and age. Scores on the Schelthen's visual rating scale of global brain atrophy was the outcome measure.

##### 2.4.3.3 Analysis of white matter abnormalities

Linear regression analysis was used to investigate the relationship between white matter abnormalities and age. The composite score on the Fazekas visual rating scale was the outcome measure (CWMA=PVA+DWMA).

#### 2.4.4 Combined cognitive and neuroimaging analyses

We used forward linear regression analyses to investigate whether age, specific frontal lesions locations (left and right lateral, medial and orbito**-**frontal), global brain atrophy and WMA predicted the discrepancy scores on the two executive tasks in frontal patients.

## 3 Results

### 3.1 Demographic and clinical results

Frontal patients, non-frontal patients and healthy controls were well matched for age (*F* (2, 162)=1.018, *p*=.363), gender (*χ*^2^ (=.475, df=2, *p*=.788), NART IQ (*F* (2, 162)=1.703, *p*=.185) and years of education (*F* (2, 162)=.181, *p*=.835). There was no significant difference between frontal and non-frontal patients in time between damage and assessment (*t* (96)=.021, *p*=.984), laterality of lesion (*χ*^2^=.331, df=1, *p*=.565) or the number of patients with hemiparesis or hemianopia (*χ*^2^=.019, df=1, *p*=.889 and *χ*^2^=2.940, df=1, *p*=.086, respectively; see Table 1).

#### 3.1.1 Effect of age on cognitive performance results

Linear regression analyses were performed to investigate the relationship between age and the discrepancy score for each cognitive test. Discrepancy scores were calculated as the difference between patients' post-morbid and estimated premorbid scores.

**Fig. 1:**
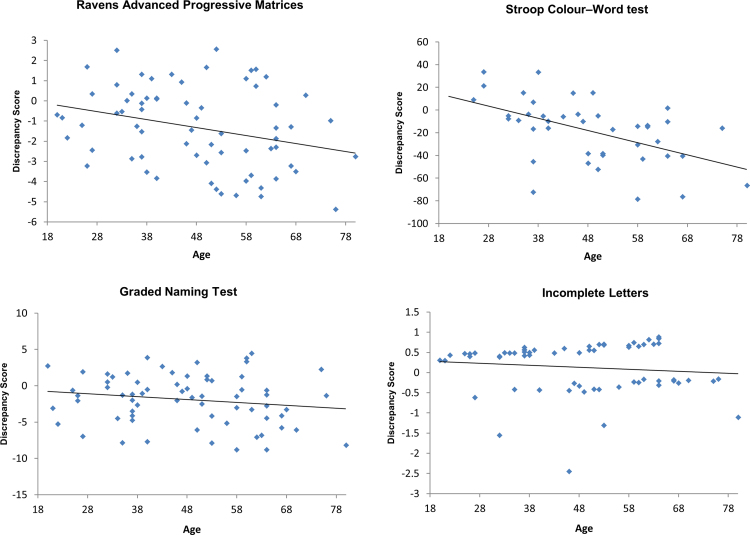
Frontal patients' discrepancy score as a function of age and the corresponding regression lines for each neuropsychological test. Legend: ◊=frontal patients, −=frontal patients regression line. Discrepancy score is reported on the *y*-axis and represents differences in absolute value for each test. 0 represents no discrepancy between each patient's measured post-morbid score on cognitive tests compared with his/her estimated premorbid score. Larger negative values reflect greater decline in performance from premorbid estimates.

In the frontal patients, we found that age significantly predicted the discrepancy score for the RAPM (*r*^2^=.084, *F* (1, 66)=6.039, *p*=.017, *α*=.05) and the Stroop test (*r*^2^=.272, *F* (1, 40)=14.958, *p*<.001, *α*=.05). In contrast, age did not predict the discrepancy score for the GNT (*r*^2^=.029, *F* (1, 66)=2.005, *p*=.161, *α*=.05) or the IL (*r*^2^=.014, *F* (1, 63)=0.884, *p*=.351 *α*=.05: see Fig. 1). In the Supplementary materials, we report the relationship between age and the discrepancy score and aetiology (i.e., stroke and tumour; see Fig. S1).

In the non-frontal patients we found that age did not significantly predict the discrepancy score on the RAPM or GNT (*r*^2^=.003, *F* (1, 43)=.124, *p*=.727, *α*=.05; *r*^2^=.008, *F* (1, 43)=.335, *p*=.566, *α*=.05, respectively; see Fig. S2. in the Supplementary materials).

#### 3.1.2 Executive performance across the three age groups results

The younger, middle-aged and older frontal patients and HC were matched for age, gender, NART and years of educations, although the older participants had fewer years of education than the younger participants. There was no significant difference in the mean time between damage and assessment across the three age groups (see Supplementary material S4 and Table S2).

We found a significant main effect of participant group on performance on the RAPM and the Stroop test with frontal patients performing significantly more poorly than HC (*F* (1, 112)=15.174, *p*<.001; *F* (2, 84)=15.605, *p*<.001, *α*=.05, respectively).

**Fig. 2:**
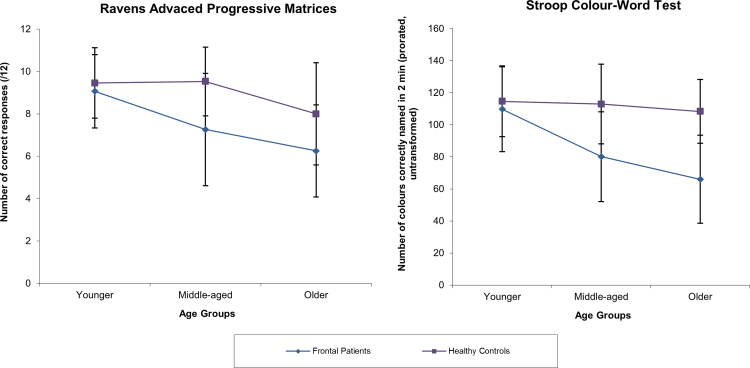
Means and standard deviations for the younger, middle-aged and older frontal patients (blue line) and healthy controls (purple line) on the executive test. Legend: error bars represent ±1 standard deviation. (For interpretation of the references to colour in this figure legend, the reader is referred to the web version of this article.)

There was also a significant main effect of age group on both tasks (*F* (2, 112)=7.907, *p*=.001; *F* (2, 84)=8.267, *p*=.001, *α*=.05 respectively). We found a marginal interaction between participant group and age group on the RAPM (*F* (2, 112)=2.917, *p*=.058, *α*=.05) and a significant interaction on the Stroop test (*F* (2, 84)=4.998, *p*=.009, *α*=.05). Post-hoc analysis revealed that older participants performed significantly more poorly than younger participants (RAPM; *p*<.001; Stroop test: *p*=.001, *α*=.017, respectively). Middle-aged participants tended to perform more poorly than younger participants on the RAPM and Stroop tests (*p*=.038 and *p*=.021, *α*=.017, respectively). Age group significantly affected the performance of frontal patients on the RAPM and Stroop test (*F* (2, 112)=11.157, *p*<.001; *F* (2, 84)=11.915, *p*<.001, *α*=.017 respectively), but not in HC (*F* (2, 112)=1.490, *p*=.230; *F* (2, 84)=0.777, *p*=.463, *α*=.017 respectively). We performed post-hoc simple effects analyses and found that middle-aged and older frontal patients performed significantly more poorly than their HC counterparts (RAPM: *p*=.001 and *p*=.01 *α*=.017, respectively; Stroop test: *p*<.001 and *p*=.005 *α*=.017, respectively) and significantly more poorly than younger patients (RAPM: *p*=.016 and *p*<.001, *α*=.017 respectively; Stroop test: *p*=.001 and *p*<.001, *α*=.017, respectively; see Fig. 2).

### 3.2 Neuroimaging results

#### 3.2.1 Frontal lesions results

**Fig. 3:**
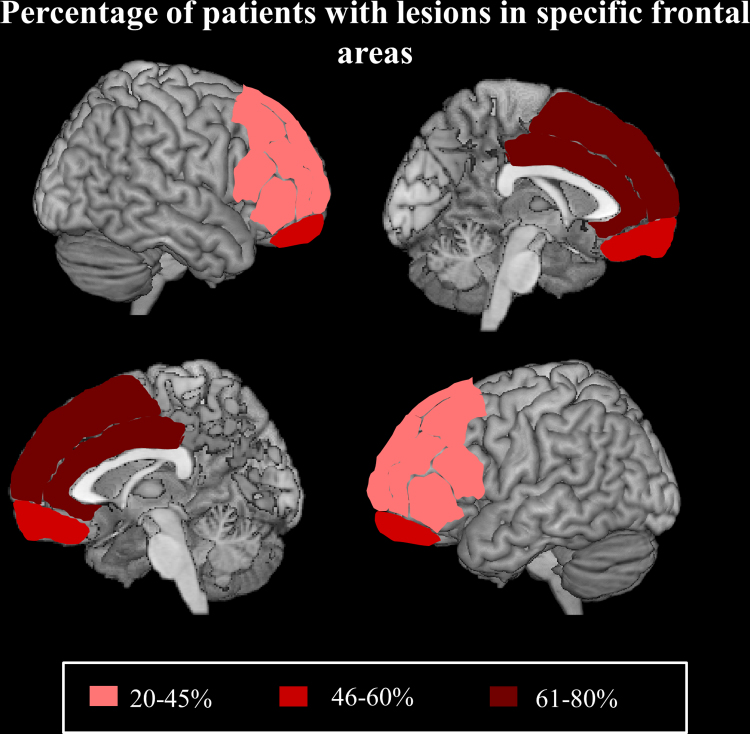
Percentage of patients with lesions in specific frontal areas projected on a standardised MNI template. Legend: shading illustrate the percentage of patients with primary and secondary damage to lateral (right and left), medial and orbito frontal regions.

T1-weigthed MRI or CT scans were available for 61 out of the 68 frontal patients. There was a significant difference in the number of patients with damage to the four main frontal areas (*χ*^2^=13.317, df=3 *p*=.004). There were significantly more patients with medial damage than patients with damage to left or right lateral areas (medial versus left lateral; *χ*^2^=11.849, df=1, *p*=.001, *α*=.0125; medial versus right lateral *χ*^2^=7.449, df=1, *p*=.006, *α*=.0125) and a non-significant trend for damage to the orbitofrontal area (medial versus orbitofrontal area *χ*^2^=5.638, df=1, *p*=.018, *α*=.0125). There was no difference in the number of patients with lesions in any of the other frontal areas (see Fig. 3).

#### 3.2.2 Global brain atrophy results

DE/FLAIR or CT scans were available for 52 out of the frontal 62 patients. Regression analysis indicated that age significantly predicted the degree of global brain atrophy as quantified by Scheltens et al.'s (1997) scale (*r*^2^=.224, *F* (1, 50)=14.396, *p*<.001, *β*=.020, *α*=.05).

#### 3.2.3 White matter abnormalities (WMA) results

The results of the regression analysis showed that age significantly predicted WMA on the composite score (CWMA) obtained by summing Fazekas's two white matter sub-scales (CWMA=PVA+DWMA; *r*^2^=.223, *F* (1, 50)=14.025, *p*<.001, *β*=.041, *α*=.05).

### 3.3 Combined cognitive and neuroimaging results

The results of the forward linear regression analyses indicated that only age significantly predicted the discrepancy scores on the RAPM (*r*^2^=.111, *F* (1, 50)=6.119, *p*=.017, *β*=−.043, *α*=.025). Both age and left lateral lesions predicted the discrepancy score on the Stroop test (*r*^2^=.45, *F* (1, 31)=12.822, *p*<.001, Age *β*=−1.24, *p*<.001; and Left lateral *β*=−25.21, *p*=.002, *α*=.025). Global atrophy, WMA and other frontal lesion locations did not significantly predicted the discrepancy scores on the RAPM or the Stroop test (*p*>.05).

## 4 Discussion

We investigated the effect of age on the cognitive performance of a large sample of patients with focal unilateral frontal or non-frontal lesion and healthy controls. We reviewed participants' data collected during a single cognitive assessment which included executive (RAPM and Stroop test), nominal (GNT) and perceptual (IL) tasks in the frontal and HC participants and an executive (RAPM) and a nominal (GNT) task in the non-frontal patients. We also reviewed the neuroimaging data available. Following the procedure originally developed by Woolgar et al. (2010), to estimate the magnitude of IQ deficit after frontal and non-frontal lesions, we found that age significantly predicted the magnitude of the impairment on the two executive tests (RAPM and Stroop test) in our frontal patients. However, age did not predict the magnitude of the impairment on the nominal and perceptual tasks. Importantly, in the non-frontal patients, age did not predict the magnitude of the impairment on the RAPM or GNT. These findings suggest that age specifically exacerbates executive impairment following frontal lesions.

Our results also demonstrated that age significantly predicted the degree of global brain atrophy and WMA, a finding consistent with previous studies in the literature (e.g., Raz et al., 2005; Elderkin-Thompson et al., 2008; Gao et al., 2011). However, our combined cognitive and neuroimaging analyses revealed that the exacerbated executive impairment was not associated with lesions to specific frontal subregions, or an increase in global brain atrophy or WMA. Only age significantly predicted the exacerbated executive impairment. The only exception was the left lateral lesions, which together with age, were associated with impairment on the Stroop task (e.g. Demakis, 2004).

Following suggestions that the effects of age on cognition and brain anatomy may manifest in middle and in older age (e.g. Deary et al., 2009), we also investigated executive performance across three age groups in our frontal patients and healthy controls. We found that middle aged and older frontal patients performed significantly poorer than their corresponding middle aged and older healthy controls as well as the younger frontal patients. Thus, it appears that the exacerbated executive impairment following frontal lesions manifests as early as from middle age. These findings add to the handful of previous studies primarily involving non-focal lesions reporting that age detrimentally impacts executive performance (e.g., Raymont et al., 2008; Alvarez and Emory, 2006; Grafman et al., 1988). For example, Senathi-Raja et al. (2010) reported disproportionately poorer executive performance in older (>55 years) than younger (35–54 years) TBI patients with respect to healthy controls.

Our results are consistent with the notion commonly reported in the literature regarding the critical role of the frontal lobes in counteracting the effects of ageing. The influential STAC model proposed that “…behaviour is maintained at a relatively high level with age, despite neural challenges and functional deterioration, due to continuous engagement of compensatory scaffolding – the recruitment of additional circuitry that shores up declining structure whose functioning has become noisy, ineffective or both…” (Park and Reuter-Lorenz, 2009, p. 10; for review see Reuter-Lorenz and Park (2014)). According to this model, healthy aging adults rely extensively on scaffolding to compensate for the decline in cognitive functioning associated with changes in brain structure, neurochemistry and functional activation. The model suggests that these brain scaffolding processes largely reside in the prefrontal cortex. Functional neuroimaging studies have repeatedly reported an age-related reduction in posterior activity coupled with increased frontal activity in healthy older adults. This posterior–anterior shift in aging (PASA) has been typically attributed to functional compensation mechanisms (e.g., Grady et al., 1994; Davis et al., 2008). Similarly the HAROLD model (Cabeza, 2002) suggests that prefrontal activity during cognitive performance tends to be less lateralised in older than in younger adults (e.g., Berlingeri et al., 2013) and this reduction in hemispheric asymmetry may be due to compensatory mechanisms or a dedifferentiation of prefrontal specialisation.

Our documented exacerbated executive decline in frontal patients, together with sparing of other cognitive abilities such as nominal and perceptual functions is in line with the view that the prefrontal cortex plays a critical role in aging to counteract cognitive and neuronal decline. We would speculate that in our middle aged and older frontal patients, the processing power available within the frontal cortex falls below the threshold needed to successfully complete executive tasks due to the combined effects of aging, frontal lesion and age-related abnormalities (e.g., Valenzuela et al., 2007; Raz et al., 2005).

Our findings are particularly relevant in the context of cognitive rehabilitation. Expensive rehabilitation programmes for cognitive impairments are becoming increasingly popular. However, to date there is a paucity of reliable markers predicting cognitive outcomes in individual patients. Previous research has indicated that executive impairments predict rehabilitation participation, post-rehabilitation functional status and long term cognitive impairment following stroke (Skidmore et al., 2010; Galski et al., 1993; Nys et al., 2005). Our study suggests that both lesion location and age can exacerbate executive impairment which, in turn, may affect long term cognitive outcomes. Thus, we would tentatively suggest that age and frontal lesions may be two variables that should be given careful consideration when weighing up inclusion in cognitive rehabilitation programmes.

As far as we are aware our study represents the first investigation of the complex relationship between age, cognitive performance and focal brain lesions in a large sample of patients with unilateral frontal or non-frontal lesions and healthy controls. Our findings are clinically relevant and contribute to the field of abnormal ageing. It is of course subject to a number of important methodological limitations. We retrospectively reviewed cross-sectional data, thus some imaging and cognitive data were missing. It has been reported that cross-sectional studies have a limited validity when investigating the relationship between age, brain and cognition (e.g. Lindenberger et al., 2011; Maxwell and Cole, 2007; Raz and Lindenberger, 2011). Moreover, our missing data may have resulted in a selection bias, although the results of our missing value analyses suggest this is not the case. Thus, we acknowledge that only limited conclusions can be drawn from our study and there remain many outstanding questions. It also remains possible that an exacerbated effect of age may occur in other cognitive domains, such as memory, and in non-frontal patients, had they being more extensively investigated.

We should also consider a possible confound linked to the cardiovascular health of our patients and healthy controls. Regrettably we did not have information available on parameters such as hypertension and genetic variants associated with increased risk of cardiovascular disease. These variables have been linked with executive performance (e.g. perseverative errors on the Wisconsin Card Sorting test) and with WMA (e.g. Raz et al., 2003). However, we investigated the effect of age on cognitive performance in our tumour and stroke frontal patients separately. We found a significant or near significant effect for the larger tumour group and a significant effect for the much smaller stroke group on the Stroop test but not on the RAPM. Of note too is the finding that the effect of age on the RAPM in the non-frontal stroke and tumour patients was far from significant. These findings suggest that the cardiovascular health of our patients and healthy controls, although an important factor, is unlikely to be a major confound in our study.

In our study we cannot disambiguate the effect that age of lesion onset can have on performance, since the interval between time of damage and cognitive assessment in our patients was rather short (on average approximately only 13 months). Similarly, we cannot ascertain the compounding effect of years of life spent with a disability. However, we do note that there was no significant difference in terms of years of living with brain damage, and the incidence of hemiparesis and hemianopia between frontal and non-frontal patients. Despite this, age only significantly exacerbated executive impairment in our frontal patients.

As our original aim was to review the cognitive data of a large sample of frontal and non-frontal patients we included patients with clinical MRI scans of differing quality or CT scans. While this allowed us to localise lesions the left or right hemisphere for all patients, these imaging methods only allowed us to further analyse the primary lesion site of our frontal patients. Ratings of global brain atrophy and white matter abnormalities could also only be undertaken for the frontal patients. Future studies should investigate longitudinally the complex relationship between age, focal lesions size and location, atrophy and WMA on a wider range of cognitive tasks.

In conclusion, we suggest that age exacerbates the effect of frontal lesions on executive functioning. In our view, it is the computational power of the relevant frontal cortical systems that is probably the most critical variable. The combined effect of frontal lesions and ageing causes the computational power to fall below the threshold needed to successfully complete executive tasks.

## Figures and Tables

**Table 1:** Frontal patients, non-frontal patients and healthy controls: demographic and clinical data.

	**Frontal *n*=68**	**Non-frontal *n*=45**	**Healthy controls *n*=52**
$\bar{x}$**Age (SD)**	47.91^*^ (14.74)	51.19^*^ (13.53)	47.42^*^ (13.70)
**Gender (Male/Female)**	38/30	24/21	26/26
$\bar{x}$**NART IQ (SD)**	109.03 (10.42)	111.69 (9.50)	112.02 (9.04)
$\bar{x}$**Years of education (SD)**	13.17 (2.90)	13.52 (3.07)	13.81 (3.33)
**Time between damage and assessment (SD)**	13.33^+^ (24.55)	13.22^+^ (27.69)	−
**Hemisphere of lesion (left/right)**	37/31	22/23	−
**Hemiparesis/Hemianopia (No)**	5/3	3/6	−

NART=National Adult Reading Test, No=number of participants, $\bar{x}$=mean, SD=standard deviation (in parentheses), *=Years, +=Months, −=Not applicable.

## Footnotes

Appendix A Supplementary data associated with this article can be found in the online version at doi:10.1016/j.neuropsychologia.2015.06.011.
