# Supplementary material for: The effect of age on cognitive performance of frontal patients
Source: Neuropsychologia. 2015 Aug;75:233–41. doi: 10.1016/j.neuropsychologia.2015.06.011 (PMC4542524; doi:10.1016/j.neuropsychologia.2015.06.011)
Supplement: Supplementary file 1 — Supplementary material [file mmc1.doc]

**Supplementary material**

**S1. Description of tests used in the Cognitive investigation**

1. The National Adult Reading Test (NART) is an irregular word reading task used as a measure of estimate optimal pre-morbid functioning (Nelson, 1982).
2. Raven’s Advanced Progressive Matrices (RAPM, Raven, 1976) is an untimed, relatively culture-free, non-verbal test of abstract reasoning, requiring the completion of a visual pattern. The total number of correct responses out of 12 was recorded.
3. The Stroop Test (Trenerry, Crosson, Deboe & Leber, 1989) is a test of response inhibition. The total number of coloured words out of 112 for which the colour was correctly named in two minutes was recorded. If a participant completed the task in less than two minutes, the raw scores were prorated to reflect the score that would have been obtained in two minutes.
4. The Graded Naming Test (GNT, McKenna & Warrington, 1980) is a test of object naming. The total number of items correctly named out of 30 was recorded.
5. The Incomplete letters subtest from the Visual Object and Space Perception Battery (VOSP IL, [Warrington & James, 1991](#_ENREF_45)) is a test of perceptual function. The total number of errors on the task was recorded (max 20).

**S2. Missing Values Analyses**

No demographic variables were missing for any participants. Thus, these variables were not analysed. Some data were missing for the neuroradiological and cognitive variables. To ensure that our results were not susceptible to systematic bias, we ran three missing values analyses. Little’s MCAR test was used to investigate if the data were missing completely at random.

**Missing Value analysis 1. Frontal patients and Healthy Controls on All Cognitive Data.** This analysis was conducted on all cognitive variables for the frontal patients and healthy controls. Little’s MCAR test indicated that there was no systematic bias in the data (χ^2^ = 10.702, df = 6, p = .098)

**Missing Value analysis 2. Frontal Patients, Healthy Controls and Non-frontal Patients on RAPM and GNT data.** This analysis was conducted on participants on the RAPM and the GNT. We did not have data for the non-frontal patients on the Stoop or IL; we did not include these variables in the analysis. Little’s MCAR test indicated that there was no systematic bias in the data (χ^2^ = 1.902, df = 2, p = .386).

**Missing Value analysis 3. Frontal Patients on all study variables.** Demographic, cognitive and neuroimaging data were analysed. Little’s MCAR test indicated that there was no systematic bias in the data set (χ^2^ = 73.092, df = 69, p = .345). These results suggest that our use of a listwise deletion method was justified.

**S3. Cognitive performance of left frontal patients, right frontal patients and healthy controls.**

Patients were subdivided into two groups based on damage to any of the nine left or right brain subregions (left and right frontal accordingly; see 2.3.1 Investigation of frontal lesions). To investigate whether left and right frontal and healthy controls were matched for age, gender, NART IQ and years of education, we used ANOVAs except for gender where chi-square test was used. To investigate whether cognitive performance differed for left and right frontal patients’, we used ANCOVA, with NART IQ and years of education entered as covariates. We found that left frontal patients, right frontal patients and healthy controls were well-matched for age (F (2, 117) = 0.645, p = .527); NART IQ (F (2, 117) = 2.448, p = .091) and years of education (F (2, 117) = 0.152, p = .859). There was a significant difference for gender (χ^2^ = 7.160, df = 2, p = .028) with males being more likely to have left frontal lesions and females more likely to have right frontal lesions (see table S1a). Analysis of the left and right frontal patients’ performance on the cognitive tasks revealed:

**RAPM.** There was a significant main effect of group (F (2, 115) = 6.296, p = .003, α = .05). Post-hoc analysis indicated that right frontal patients performed significantly more poorly than HC (p =.005, α = .0125) whilst there was a non-significant trend for left frontal patients (p =.028, α = .0125). There was no difference in performance between left and right frontal patients (p > .99).

**Stroop Test.** There was a significant main effect of group (F (2, 87) = 5.833, p = .004, α = .05). Post-hoc analysis indicated that left frontal patients performed significantly more poorly than HC (p = .004, α = .0125). There was no significant difference in performance between right frontal patients and healthy controls or between left and right frontal patients (p = .242 and p > .99, α = .0125, respectively)*.*

**GNT.** There was a significant main effect of group on the GNT (F (2, 115) = 3.914, p = .023 α = .05). Post-hoc analysis indicated that there was a non-significant trend for the left frontal patients perform more poorly than HC (p = .037, α = .0125). There was no significant difference between right frontal patients and HC or between left and right frontal patients (p = .122 and p > .99, α = .0125, respectively).

**IL.** There was no significant main effect of group on the IL (F (2, 111) =1.678, p = .4191 α = .05).

NART premorbid IQ significantly affected performance on GNT (F (1, 115) = 67.367, p < .001) and there was a trend towards a similar effect on the Stroop (F (1, 87) = 3.339, p = .071). Years of education significantly affected performance only on the RAPM (F (1, 115) = 18.693, p <.001).

**Table S1a. Demographic and clinical characteristics for left and right frontal patients and healthy controls.**

|  |  | **Left Frontal**  **n = 37** | **Right Frontal**  **n = 31** | **Healthy Controls**  **n = 52** | |
| --- | --- | --- | --- | --- | --- |
| **x Age (SD)** | | 46.14^*^  (13.06) | 50.03^*^  (16.51) | | 47.42^*^  (13.70) |
| **Gender (Male/ Female)** | | 26/11 | 12/19 | | 26/26 |
| **x NART IQ (SD)** | | 107.43  (10.69) | 110.94  (9.93) | | 112.02  (9.04) |
| **x years of Education (SD)** | | 14.05  (2.96) | 13.65  (2.86) | | 13.81  (3.33) |
| **Time between damage and assessment (SD)** | | 9.33 (16.06) | 17.78 (31.18) | | - |

**Table S1b.** Cognitive results for left and right frontal patients and healthy controls.

|  |  | **Left Frontal**  **n = 37** | **Right Frontal**  **n = 31** | **Healthy Control**  **n = 52** |
| --- | --- | --- | --- | --- |
| **x RAPM /12 (SD)** | | 7.92  (2.41) | 7.65  (2.52) | 9.17  (1.90) |
| **x Stroop prorated score (SD)** | | 86.11  (29.74) | 95.48  (35.12) | 112.74  (22.32) |
| **x GNT /30 (SD)** | | 19.43  (4.12) | 20.65  (4.74) | 22.40  (3.55) |
| **x IL errors/20 (SD)** | | 0.35  (0.60) | 0.55  (0.57) | 0.65  (0.69) |

*^Legend^:* NART=National Adult Reading Test, * = years, **x** = mean and SD= standard deviation (in parentheses). RAPM = Raven’s Advanced Progressive Matrices, GNT = Graded Naming Test, IL= Incomplete Letters, - = Not applicable.

**S4. Executive Performance Across Three Age Groups: Demographic and clinical characteristics**

The three age sub-groups of frontal patients and HC were matched for age (younger: t (51) = -0.619, p =.539; middle-aged: t (38) = -0.795, p =.431; and older: t (25) = 0.923, p =.365) and gender (younger: χ^2^ (1, n= 53) = 0.01, p = .974; Middle-aged: χ^2^(1, n = 40) = 1.125, p = .460; and older: χ^2^(1, n = 27) = 0.054, p = .816). Two 2 x 3 ANOVA were used to compare NART IQ scores and years of education between patients and healthy controls across the age groups. The results indicated no significant main effects or interaction for NART IQ (age group: F (2, 114) = 0.28, p = .756; participant group: F (2, 114) = 3.343, p = .070; interaction; F (2, 114) = 0.409, p = .666). For years of education, there was a significant main effect of age group (F (2, 114) = 3.523, p = .033) indicating that older participants had fewer years of education than the younger participants (p = .042). There was no main effect of participant group (F (2, 114) = 0.31, p = .579) and no interaction (F (2, 114) = 1.371, p = .258). The three age groups of frontal patients were matched for time between damage and assessment (F (2, 114) = 1.371, p = .258; see table S2). There was a significant effect of NART on RAPM and the Stroop (F (1, 112) = 5.205, p = .024; F (1, 85) = 11.797, p = .001, respectively). There was a significant effect of years of education on the RAPM (F (1, 112) = 13.014, p < .001).

**Table S2.** Demographic, clinical and cognitive results of the younger, middle-aged and older frontal patients and healthy controls.

|  | **Frontal Patients**  **n = 68** | | | | **Healthy Controls**  **n= 52** | | |
| --- | --- | --- | --- | --- | --- | --- | --- |
|  | **Younger**  **20-45** | | **Middle-aged**  **46-60** | **Older**  **61-80** | **Younger**  **20-45** | **Middle-aged**  **46-60** | **Older**  **61-80** |
|  | **n=29** | **n=23** | | **n=16** | **n=24** | **n=17** | **n=11** |
| **x Age (SD)** | 33.48^*^ (6.65) | 52.91^*^ (4.68) | | 66.88^*^ (5.66) | 34.63^*^ (6.74) | 54.06^*^  (4.25) | 65.09^*^ (3.56) |
| **Gender (Male/Female)** | 18/11 | 12/11 | | 8/8 | 15/9 | 6/11 | 5/6 |
| **x NART IQ (SD)** | 109.31 (9.36) | 108.70 (11.59) | | 109.00 (11.17) | 110.67 (8.93) | 112.29 (10.33) | 114.55 (7.16) |
| **x years of Education (SD)** | 14.55 (2.76) | 13.13 (3.22) | | 13.69 (2.52) | 14.67 (2.73) | 13.82 (3.75) | 11.91 (3.33) |
| **Time between damage and assessment (SD)** | 13.57^+^ (26.85) | 15.42^+^ (27.45) | | 9.21^+^ (12.80) | - | - | - |
| **x RAPM /12 (SD)** | 9.07 (1.73) | 7.26 (2.65) | | 6.25 (2.18) | 9.46 (1.67) | 9.53 (1.63) | 8.00 (2.41) |
| **x Stroop prorated score (SD)** | 109.77 (26.51) | 80.09 (28.02) | | 66.00 (27.46) | 114.68 (22.10) | 112.93 (24.91) | 108.37 (19.87) |

*^Legend^:* NART=National Adult Reading Test, No=number of participants, **x** = mean, SD= standard deviation (in parentheses). * = Years, + = Months, - = Not applicable, RAPM = Raven’s Advanced Progressive Matrices.

**S5. Effect of age on executive performance across different aetiologies**

It is well known that cardiovascular health factors, such as hypertension, are associated with poor performance on executive tasks (e.g. Raz, et al., 2003). Thus, it may be that the exacerbated effects of age we documented in frontal patients is driven our stroke patients. To investigate this further, we subdivided our frontal sample according to aetiology: tumour (n=51) and stroke (n=17). We used the procedure adopted in our primary analysis (The Effect of Age on Cognitive Performance). We investigated the relationship between age and discrepancy measures on the RAPM and Stroop test in tumour and stroke patients independently. Independent samples t-tests were also used to examine if there was a difference between tumour and stroke patients in the degree of global atrophy and white matter abnormalities. In tumour patients we found that there was a significant effect of age on the discrepancy score of the Stroop task (r^2^ = .190, F (1, 30) = 6.816, p = .014, α = .025) and a trend towards significant on the RAPM (r^2^ = .075, F (1, 50) = 3.980, p = .052, α = .025). In stroke patients we found that there was a significant effect of age on the discrepancy score on the Stroop task (r^2^ = .600, F (1, 10) = 13.478, p = .005, α = .025), but not the RAPM (r^2^ = .123, F (1, 16) = 2.110, p = .167). Strikingly the regression lines for tumour and strokes patients on both tasks were very similar (see Figure S1). Furthermore we found no significant difference between tumour and stroke patients in global atrophy and white matter abnormality ratings (t (50) = -0.402, p = .690 and t (49) = -1.168, p = .248, respectively).

**Figure S.1.** Discrepancy scores for stroke and tumour frontal patients and corresponding regression lines on the two executive tests.


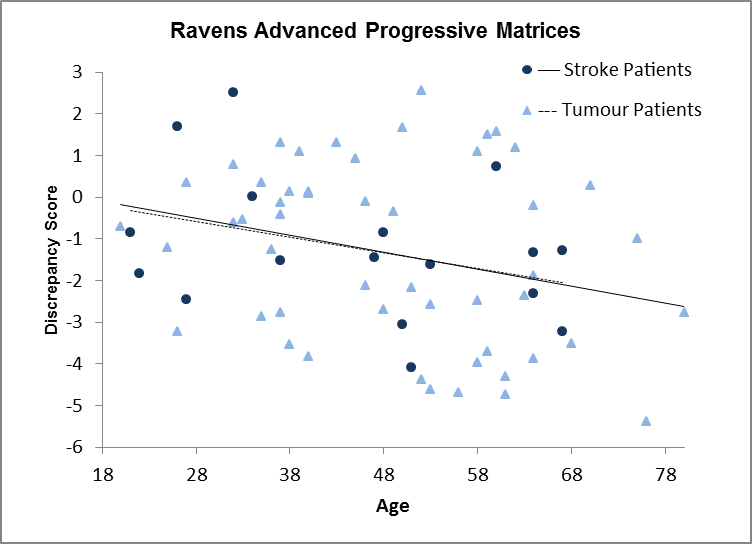

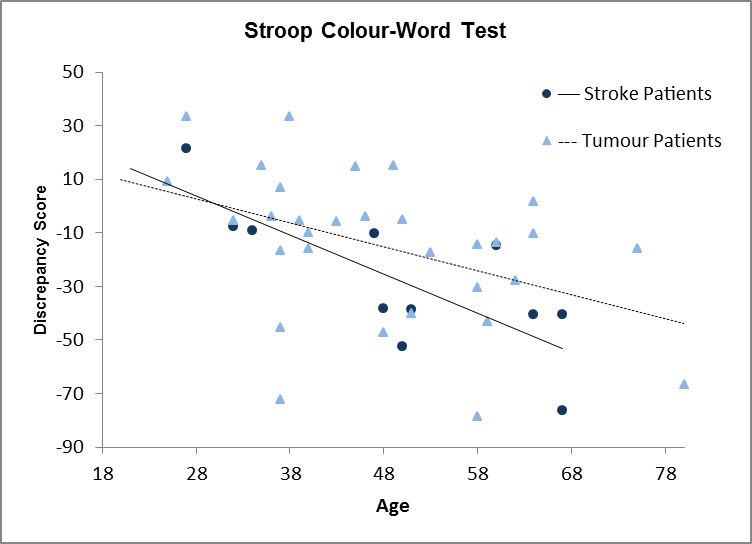


Legend. Δ = frontal tumour patients, • = frontal stroke patients -- = tumour patients regression line, ― = stroke regression line.

Legend: Δ = frontal tumour patients, • = frontal stroke patients, -- = tumour patients regression line, ― = stroke regression line.

**Figure S.2.** Non-frontal patients’ discrepancy score as a function of age and the corresponding regression lines for the cognitive tests.


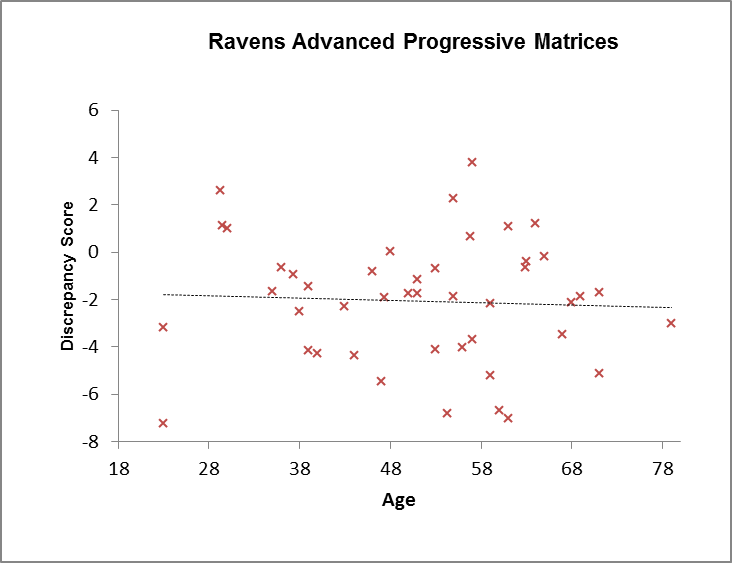

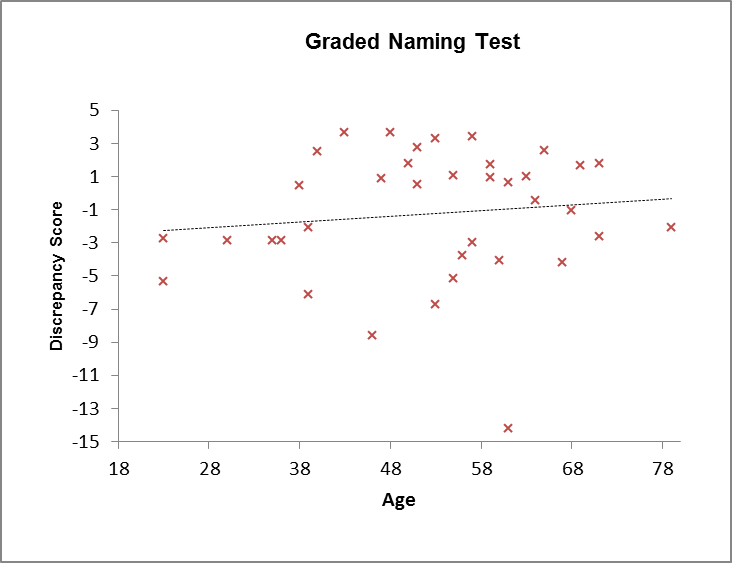


Legend: x = non-frontal patients, -- = non-frontal patients regression line

Legend: x = non-frontal patients, -- = non-frontal patients regression line.
